# Grandparental co-residence and grandchild survival: the role of resource competition in a pre-industrial population

**DOI:** 10.1093/beheco/arad013

**Published:** 2023-03-27

**Authors:** Simon Chapman, Mirkka Danielsbacka, Antti O Tanskanen, Mirkka Lahdenperä, Jenni Pettay, Virpi Lummaa

**Affiliations:** Department of Social Research, University of Turku, Turku, Finland; Department of Social Research, University of Turku, Turku, Finland; Population Research Institute, Helsinki, Finland; Department of Social Research, University of Turku, Turku, Finland; Population Research Institute, Helsinki, Finland; Department of Biology, University of Turku, Turku, Finland; Department of Social Research, University of Turku, Turku, Finland; Department of Biology, University of Turku, Turku, Finland

**Keywords:** Finland, grandchildren, grandmother hypothesis, grandparents, resource competition, sex specific reproductive strategies, three-generational household

## Abstract

Although grandparents are and have been important alloparents to their grandchildren, they are not necessarily only beneficial but can also compete with grandchildren over limited resources. Competition over parental care or other resources may exist especially if grandparents live in the same household with grandchildren and it can be dependent on grandchild age. By utilizing demographic data collected from historic population registers in Finland between 1761 and 1895 (study sample *n* = 4041) we investigate whether grandparents living in the same household with grandchildren are detrimental or beneficial for grandchild survival. Having a living but not co-residing grandmother or grandfather were both associated with better survival whereas having a co-resident grandfather was associated with lower chance to survive for infants (age < 1 year). Separating the effect between maternal and paternal grandparents and grandmothers and grandfathers revealed no differences in the effects between lineages. Negative effect of having a co-residing grandfather was not significant when grandfathers were separated for lineage specific models. These results implicate that accounting for the co-residence status and child’s age, grandparents were mostly beneficial when not co-residing with very young children and that having a co-residing grandfather at that age could be associated with lower chances to survive. Predictions made by grandmother hypothesis and resource competition both received support. The results presented here also offered comparison points to preindustrial and contemporary three-generational families.

## Teaser

Grandparents, grandmothers especially, have shown to be beneficial for grandchild survival in pre-industrial societies. Our results from pre-industrial Finland indicate that living with grandparent is not necessarily beneficial and having a co-residing grandfather is actually associated with lower chances to survive for infants. This could be due to the competition over parental care or other resources between grandparent and grandchild experienced in three-generational households.

## INTRODUCTION

Grandparents have been important alloparents in non-industrialized or “traditional” populations and still are in present-day societies (Coall and Hertwig 2010). They invest various ways in grandchildren and these investments include all time, care, knowledge, and resources grandparents provide to their descendants. Such grandparental investment is associated with grandchild development and wellbeing (Sear and Coall 2011). In traditional and historic populations grandmother presence (that has frequently been a proxy for investment) has been associated with both a greater probability of surviving childhood (Sear and Mace 2008; Beise and Voland 2002; Beise 2005; Lahdenperä et al. 2004; Sear and Coall 2011) and better nutrition (Hawkes et al. 1998; Sear et al. 2000).

The beneficial effect of grandmothers has been explained by the grandmother hypothesis which predicts that in fact, post-reproductive life of human females is the outcome of the adaptive benefits gained by investing in the reproductive efforts of own children and grandchildren (Hawkes et al. 1998). Although the grandmother hypothesis has been challenged and it is unlikely the only explanation for the evolution of reproductive cessation in the first place (Cant and Johnstone 2008), helping offspring may still have accounted for the length of post-reproductive life in human females (e.g., Lahdenperä et al. 2004). The context-dependence of grandmother effects has been emphasized in recent studies (e.g., Chapman et al. 2019; Sheppard and Sear 2016) and it has been questioned whether the grandparents, in every contexts, improve survivorship.

Namely, grandparents may not necessarily be only beneficial for their grandchildren either in contemporary or historic societies. Studies concerning the effects of grandparental investments for child development and well-being in contemporary societies have provided mixed results (Sadruddin et al. 2019; Tanskanen and Danielsbacka 2018). The association between grandparental investment and grandchild wellbeing is likely to differ especially between non-coresiding, three-generational, and custodial grandparent families (Sadruddin et al. 2019; Tanskanen and Danielsbacka 2019). Based on prior studies, child outcomes in present-day three-generational and custodial grandparent families are usually poorer than in intact families with no co-residing grandparent because of the social selection of single parent and socially disadvantaged families into a custodial grandparent or three-generational living arrangement (e.g., Dunifon et al. 2014; Kreidl and Hubatková 2014; Pittman 2007).

Previous studies from historical and traditional societies have found that associations of grandmaternal presence and grandchild outcomes can vary under different contexts and indicated the existence of age and lineage-specific grandmother effects (e.g., Chapman et al. 2019; Sheppard and Sear 2016; Beise and Voland 2002; Willführ et al. 2022; Hacker et al. 2021). Indeed, in traditional and historic populations grandmother presence has even been associated with negative grandchild outcomes, i.e., decreased probability to survive (Voland and Beise 2002; Strassmann 2011), particularly in the case of paternal grandmother presence (Chapman et al. 2019; Lahdenperä et al. 2012; Sheppard and Sear 2016; Strassmann et al. 2006; Voland and Beise 2002). Perhaps, the most important explanation for negative associations with grandparents, especially paternal ones, is the resource competition over parental care and other resources between grandparents and grandchildren (Strassmann 2011), which may occur particularly when grandparents are living in the same household with grandchildren. In addition, the more people there are in the same household, the bigger is the risk for infectious deceases. Relative severity of infectious deceases is highest in infants and in older age groups (Glynn and Moss 2020) which is why the presence of grandparents in the same household could also increase the mortality of infants.

Resource competition occurs when individuals compete with one another over limited resources (e.g., food, shelter) (Strassmann 2011). Whilst humans are cooperative breeders (Hrdy 1999; 2009), it does not preclude them from competing with their kin (Alexander 1974; Cant and Johnstone 2008; Ji et al. 2014). In historical and traditional populations, where the maintenance of older family members was usually the responsibility of their adult children, the grandparents could actually have had a detrimental effect on grandchild survival because grandparents who lived in the same household with grandchildren might have competed with them over the same resources which could have been food but also parental time and care (Chapman et al. 2019; Strassmann and Garrard 2011; Strassmann 2011). As they age, grandparents switch from being net producers of resources to net consumers (Kaplan et al. 2000; Hill and Hurtado 2009; Hooper et al. 2015), and this could exacerbate within-family resource competition if the grandparents are co-resident. Because the old couple usually co-resided with the oldest son and his family in patrilocal Finland studied here (Gaunt 1983; Moring 2003), paternal grandparents in particular could be harmful or compete over household resources with their grandchildren.

Another theoretical explanation for the potential detrimental effect of paternal grandparents are sex-specific reproductive strategies (Beise and Voland 2002; Voland and Beise 2005). Producing and raising a single descendant is more costly for women (due to pregnancy and lactation) than for men, meaning also that maternal investment is more obligatory than paternal investment (Trivers 1972). This has been claimed to result in different reproductive strategies for men and women and also between maternal and paternal relatives (Euler 2011; Perry and Daly 2017; but see Moya et al. 2016). Sex-specific reproductive strategies can be used to explain the potentially different effects of maternal and paternal grandparents on child outcomes.

Due to lower obligatory levels of paternal than maternal investment, men can theoretically increase their fitness more than women by mating with several partners (but see Kokko and Jennions 2003). As for grandparents, maternal grandparents are related to the reproductive woman in a partnership, whereas paternal grandparents are not. Thus, it is more important for maternal than paternal grandparents to protect the health and wellbeing of women and their children (Perry and Daly 2017). In contrast, from the perspective of paternal grandparents’ fitness, it may not be as important as for maternal grandparents to protect the health and wellbeing of mother which can lead to harmful effects on already existing children (Mace and Sear 2005).

Finally, reproductive conflict between grandmother and mother may exist if they are reproducing simultaneously. Reproducing at the same time may have detrimental effects for child survival, especially in harsh environments including historical Finland (Pettay et al. 2016). The severity of the reproductive conflict is related to the degree of relatedness between the females and their offspring and it is more likely to occur between unrelated females (e.g., mother-in-law and daughter-in-law) (Cant and Johnstone 2008). However, the effect of reproductive conflict in three-generational households studied here, is likely to be very minor because there are only few women from different generations that do reproduce simultaneously (Lahdenperä et al. 2012) and that is why we are not testing it per se.

In line with sex-specific reproductive strategies and resource competition, in 18th and 19th century Finland (Chapman et al. 2019; Chapman et al. 2021; Lahdenperä et al. 2004) and in 18th and 19th century Germany (Beise and Voland 2002), having maternal grandmothers have been found to be beneficial for grandchild survival but paternal grandmothers, especially when they are old or close to death (Chapman et al. 2019), have found to be detrimental for grandchild survival. Reason for such differing effects could be twofold. First, the paternal grandmothers may have lived with their grandchildren and thus competition over parental care or other resources was more likely with them than with maternal grandmother. Alternatively, due to some other reason than co-residing, aging paternal grandmothers were more harmful for grandchildren than maternal grandmothers.

There is less information available on whether grandfathers’ presence might have positive or negative associations with grandchild survival in historical or traditional societies. One reason for this is that, especially in historical populations, grandfathers have had much less shared years with grandchildren than grandmothers (Chapman et al. 2017). Despite fewer studies on possible grandfather than grandmother effects, the pattern across populations appears to be that grandfather presence is not often associated with increased survival of grandchildren (Sear and Mace 2008; but see Du at al. 2022). Studies with data from historical Germany (Kemkes-Grottenthaler 2005), historical Italy (Derosas 2002), and pre-industrial Japan (Jamison et al. 2002) have actually reported negative effects of grandfathers, especially paternal grandfathers, on grandchild survival. In pre-industrial Finland, grandfather presence was not associated with increases in either the survival or fertility domains, and fitness gains from long life were negligible even when widowed and remarrying with fertile women (Lahdenperä et al. 2007; 2011). Indeed, long living grandfathers might have even been detrimental for grandchild survival (Lahdenperä et al. 2007). That said, however, a recent study from a pastoralist community in western China found that grandfathers’ presence was associated with reduction in child mortality (from ages 0 to 5 years) (Du et al. 2022). Du et al. (2022) concluded that this might be due to the fact that because of socioeconomic changes in livelihood, older men more likely than older women were economically inactive and took the alloparental child care responsibilities.

A major limitation in most previous studies is that they have not been able to take into account the co-residing status of grandparents (but see Hacker et al. 2021; Willführ et al. 2022). Instead, they address the grandmother or grandfather effect by comparing children with dead versus alive grandparents, without necessarily knowing whether the grandparent was interacting with the grandchild or not. Here, we study the effect of co-residence with grandparents on the survival of grandchildren, using demographic data collected from historic population registers in mostly patrilocal Finland and with detailed information on the residential patterns of both grandparents and grandchildren. We derive our hypotheses from theories of resource competition and sex-specific reproductive strategies. In addition, we utilize predictions made by grandmother hypothesis. As resource competition should be most severe in three-generational households we may expect that

H1) Co-residing with a grandparent(s) has detrimental effects for grandchild survival.

Not all children in three-generational households lived with their paternal grandparents though, and may have co-resided with maternal grandparents for reasons outlined below. If there is a difference in grandchild survival outcomes according to whether the co-residing grandparent was maternal or paternal, then the explanation could be derived from sex-specific reproductive strategies. Thus, our second hypothesis predicts that

H2) Co-residing with a paternal grandparent(s) is more detrimental for grandchild survival than co-residing with a maternal grandparent(s).

Since previous studies highlight grandmaternal effects (both negative and positive), and there is much less studies concerning grandpaternal effects, we have done all analyses separately for grandmothers and grandfathers. According to sex-specific reproductive strategies, grandmother hypothesis and previous evidence on grandparent effects on child survival, grandmothers in general could be more beneficial in terms of child survival than grandfathers. Hence, we predict that

H3) Co-residing with a grandfather is more detrimental for grandchild survival than co-residing with a grandmother.

The novelty of the present study comes from the fact that, for the first time, we are able to reliably study the grandmaternal and grandpaternal co-residence effect in a pre-industrial population by comparing co-residing and non-coresiding grandmother-grandchild and grandfather-grandchild pairs, and, in addition, testing the co-residence effects for maternal and paternal grandparents. Also, because previous studies have found that grandmaternal effects could be dependent on grandchild age (e.g., Chapman et al. 2019, 2021; Beise and Voland 2002), we have here studied the interaction of co-residence status and grandchild’s age.

Our study population is suitable for addressing the hypotheses above because it has variations regarding childhood mortality and grandparental co-residence. Our study period of 1761–1895 pre-dates the bulk of industrialization in Finland (Hjerppe 1989) and the subsequent onset of the demographic transition - childhood mortality rates did not immediately decline (Scranton et al. 2016). Though childhood mortality rates were high in the pre-industrial era (Chapman et al. 2018a; Pettay et al. 2016), those surviving to adulthood had a life expectancy of over 60 years (Griffin et al. 2018). The average shared time of grandmothers and grandchildren was less than 10 years during the study period (Chapman et al. 2018a), whilst the shared time with grandfathers was comparatively lower, on average below 5 years during the study period (Chapman et al. 2017).

In pre-industrial Finland - as well as in other Nordic countries and in central Europe - a common arrangement between farmer generations was a form of early inheritance (“syytinki” in Finnish) (Gaunt 1983; Kivialho 1927; Moring 2003). In this system of peasant retirement an aged farmer would give over his property in his lifetime to a relative, generally his own child, in return for food and lodging for the rest of his days (effectively a pension contract for the old people). For grandchildren, this meant that some of them were living with their grandparents in three-generational households and some were not, potentially leading to both differential grandparental investment and resource competition. Typically, the oldest son inherited the farm, so co-resident children were most likely living with their paternal grandparents. But there were exceptions: If an old couple did not have any sons or the sons were for whatever reason unwilling or incapable to take on the farm, the farm was inherited by a daughter and a son-in-law (Gaunt 1983; Moring 2003). Old couples usually retired at the age between 50 and 60 in the 18th and 19th centuries and during that period the retirement age rose steadily being closer to 60 at the beginning of 20th century (Gaunt 1983).

It has been estimated that the prevalence of three-generational households in 1700–1800 south-western Finland was about 20–30 percentage of households (Moring 2003). These households consisted of parents with married children. Household structure was heavily dependent on local economy. Three-generational households and more complex households were common in regions where land or the accumulation of capital was needed for economic activity (Moring 2003). During the 19th century the increase in landless population decreased the share of multigenerational households because landless and households with scarce resources did not have multiple generations or other extended family living under the same roof in the same extent as farmers (Moring 2003, 2016).

Syytinki in pre-industrial Finland concerned only fairly wealthy farmers, those who owned their farm, and thus probably had more resources than the poorest agrarian population (tenant farmers, crofters etc.). However, because Finland was a relatively poor country, either the landowner or peasants were not as wealthy as they might have been in some other countries at the same time. In any case, the context of pre-industrial Finland is different from early 1900 century Sweden or early 1900 century US (Hacker et al. 2021; Willführ et al. 2022). Both of the latter mentioned societies were more alike contemporary societies with nuclear families where three-generational households are rare among well off people and more common among people on lower socioeconomic standings. Three-generational households were in pre-industrial Finland common among reasonably wealthy farmers whereas nuclear families were more common among poorer groups such as landless, crofters, tenant farmers etc. (Moring 2003).

## MATERIAL AND METHODS

### Data and variables

We investigated the effect of co-residence with grandparents on the survival of grandchildren, using demographic data collected from historic population registers in Finland. These registers were maintained by the Lutheran Church, as required by law, and detail, e.g., births, deaths, children, marriages, household compositions, and occupations. From these, we were able to construct the full life-histories of many individuals and their descendants. The dataset used for this particular study comes from six pre-industrial parishes from across Finland (Southwest Finland: Rymättylä, Hiittinen, Pirkanmaa, Ikaalinen; Northern Ostrobothnia: Pulkkila; Karelia: Rautu, Jaakkima), with records from the 18th and 19th centuries (1761–1895). Data from church records are publicly accessible through the institutions that manage these data, i.e., Finnish Genealogical Society and the National Archives of Finland. Because the data is historic i.e., people it concerns have lived in 18th and 19th centuries, there has been no need for ethical approval for the study.

Individuals were categorized into two social classes on the basis of their father’s occupation: landed and landless. These social classes have previously been found to capture variation between individuals in mortality, birth rate, and marriage patterns (Pettay et al. 2007). Co-residence was defined by whether the grandchild’s childhood home (i.e., the recorded home of their parents) was the same as the grandparent’s recorded household. The co-residence variable was created as follows: if the grandchild was co-resident with a set of grandparents (including if only one grandparent in the set was still alive), they were marked as co-resident. If all grandparents were deceased, the variable level was “no grandparent.” If the grandchild did not live in the same household as either grandparent set but one of grandparents was living (elsewhere) they were recorded as not co-resident. Thus, we have in our sample only those children and grandparents who have recorded house name in the data meaning that we can identify with the house name variable whether they lived in the same household or not. Not all people in the dataset have recorded house name and thus they are not included in the analyses. This could potentially cause a bias in our sample but it does not. Comparing the sample with and without housing information, the proportion of landed and landless in these two groups is almost the same (no house info 48% landed; with house info 52% landed). Although there is slightly more landed households in the sample with recorded house name in the data, we can consider our sample to be sufficiently representative for the whole population.

### Statistical analysis

To analyze the survival effects of co-residence on the grandchild, we used event history analysis with a discrete time-event framework, implemented as binomial generalized mixed-effects models with a logit link function using the function glmer from package lme4 in statistical software R (Bates et al. 2015). These allow variables to change through time, such as which grandparents were alive each year or not. Focal grandchild survival was coded as 1 if they lived in a given year, or 0 if they died in that year. If an individual did not have a recorded date of death, they were censored at the last date they were known to be living. Individuals were also censored if their co-resident grandparent disappeared from the records or died before the grandchild reached age 5. Where applicable, statistical significance was considered at the level of α = 0.05. Significance of interactions, reported as chi-square tests, were obtained using likelihood ratio tests implemented with the mixed function from R package afex v0.21-2 (Singmann et al. 2017).

Our first set of models were concerned with investigating the effect of co-residence in general (H1), independent of which grandparents (mother’s parents or father’s parents) the grandchild was living with. Our main variable of interest was the interaction of age of the grandchild (continuous) with whether the child was co-resident with a grandmother (3-level factor: co-resident [*n* = 1294], living but not co-resident [*n* = 1872], no living grandmother [*n* = 1144]) or grandfather (3-level factor: co-resident [*n* = 1020], living but not co-resident [*n* = 1426], no living grandfather [*n* = 1796]). No living grandparent was included as a separate level in the variables rather than being included within “not co-resident” as a grandparent not living with their grandchildren may still provide help whilst a dead grandparent cannot – combining these levels could erroneously mask possible grandparental associations with grandchild survival. We included the interaction between age of child and co-residence status to account for possible differences in the effect of co-residence on grandchild survival by grandchild age – for example, infant survival in this population was lower when old paternal grandmothers were alive (Chapman et al. 2019), which may have arisen due to co-residence.

As previous work has shown there to be associations between grandmother lineage and grandchild survival (Chapman et al. 2019, 2018b, 2021), we then ran a second set of models for grandmothers (H2) partitioning the co-residence into maternal and paternal co-residence: (4-level factor: co-resident maternal grandmother [*n* = 279], co-resident paternal grandmother [*n* = 713], living but not co-resident grandmother [*n* = 2232], no living grandmother [*n* = 1144]) and grandfathers (H2) (4-level factor: co-resident maternal grandfather [*n*= 192], co-resident paternal grandfather [*n* = 428], living but not co-resident grandfather [*n* = 1870], no living grandfather [*n* = 1796]). We also conducted the H2 set of analyses with data that were split according to lineage, e.g., the co-residence variable in the maternal grandmother model had three levels: no living maternal grandmother, co-resident with maternal grandmother, not co-resident with maternal grandmother.

In all models, we controlled for the number of co-residing highly-dependent siblings (i.e., under the age of 5), social class (2-level factor: landed, landless), birth order of the grandchild, twinning status, grandchild sex, region of Finland (4-level factor: archipelago, central mainland, Northern Ostrobothnia [province situated to the south of Lapland], Karelia [eastern province located in present day Russia]), and mother survival status (3-level factor: alive, dead, censored). Birth cohort (in 10-year bins), accounting for social change through time, and grandmother or grandfather ID, accounting for similarities between relatives, were coded as random effects. As divorce was forbidden by the church (Sundin 1992), marriages were stable, and it does not affect the results if we use a maternal or paternal grandparent ID. After constructing this “full” model, we then used the function drop1 to sequentially remove each term. We retained terms that increased the Akaike Information Criterion (AIC) by more than 2, and removed any term that did not, to avoid overfitting the model. Terms removed through this process included: region of Finland, sex of the focal child, and social class. Although sex of the focal child could be relevant from the viewpoint of the X-linked grandmother hypothesis (Fox et al. 2010), it has been previously shown that in this study population grandmother effects do not seem to be moderated by the gender of the child (Chapman et al. 2018b). All statistical analyses were performed with R statistical software (R Core Team 2018).

## RESULTS

At birth, most individuals in the study sample had at least one living grandparent (85.9%). Of these, almost half co-resided with a grandparental set (40.1%). Co-residence was skewed towards patrilocality – 72.7% of households with intergenerational co-residence were patrilocal (almost one third of all grandchildren with at least one living grandparent co-resided with the paternal lineage). Of landed individuals, 51.8% of those with living grandparents lived in a multi-generational household, 74.9% of whom co-resided with their paternal grandparents. For landless individuals, the situation was rather different: only 26.8% of individuals with living grandparents lived in a multi-generational household, 67.5% of whom co-resided with paternal grandparents.

Looking at co-residing grandparent characteristics, of co-residing maternal grandmothers 59.7% were landed and figures were 54.9% for maternal grandfathers, 64.5% for paternal grandmothers, and 62.9% for paternal grandfathers who co-resided with a grandchild. Number of dependent siblings in the same household was highest (1.75 ± 0.72) when no grandmother was living in the same household and lowest (1.63 ± 0.70) when co-residing grandparent was maternal grandmother.

**Figure 1:**
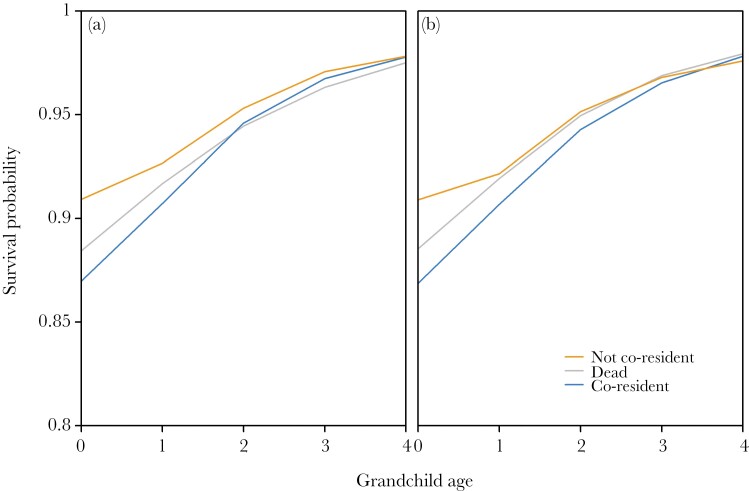
**Model-predicted grandchild survival by the interaction of grandparental co-residence and grandchild age for grandmothers/grandfathers.** Orange = both grandmothers/grandfathers are not co-resident, grey = grandmothers/grandfathers are dead, blue = a grandmother/grandfather is co-resident. Lines are smoothed splines created with the *smooth.spline* function in R, with default parameter values. a) Grandmothers, b) grandfathers. For density distributions of predicted survival probabilities, see Figure S1.

Of those individuals included in these analyses, slightly over a quarter died before reaching age 5 (26.5% in the grandmother analyses, 26.7% in the grandfather analyses), with the majority (70.2% in the grandmother analyses and 70.8% in the grandfather analyses) dying before the age of 2. Hypotheses 1 predicted that co-residing grandparent could be detrimental for grandchild survival and that the effect could be somewhat different for grandmothers and grandfathers. There was a significant interaction between grandmother co-residence status and grandchild age (χ^2^_2_ = 9.10, *P* = 0.011), indicating that there was an effect of co-residence on grandchild survival and that this effect depended on grandchild age. Though there was no difference between the grandchild age interaction with no living grandmother and a co-resident grandmother, there was a significant difference between when there was no living grandmother and when there was a living, not co-resident grandmother (β = −0.197 ± 0.068, *P* = 0.004; Table 1; Figure 1), with Figure 1a indicating that it was beneficial for very young grandchildren (age < 1) to have no co-resident grandmother. At later ages, there does not appear to be a difference.

**Table 1:** Generalized linear mixed-effect model outputs for grandchild survival by grandmother co-residence. Reference level for co-residence was “No living grandmother.” *N* is number of individuals

Fixed Effects	Estimates	Standard Error	*Z*	*P*	*N*
Intercept	2.296	0.219	10.50	<0.001	1144
Age	0.404	0.053	7.68	<0.001	
Co-resident (Yes)	0.067	0.129	0.52	0.606	1294
Co-resident (No)	0.284	0.125	2.28	0.023	1872
Living Siblings	1.151	0.038	30.04	<0.001	
Twin	−1.353	0.170	−7.95	<0.001	
Birth Order	−0.425	0.017	−25.10	<0.001	
Age * Co-resident (Yes)	−0.071	0.072	−0.99	0.325	
Age * Co-resident (No)	−0.197	0.068	−2.91	0.004	
Random Effects	Variance	Standard Deviation			
MGM ID	<0.001	<0.001			
Birth Cohort	0.010	0.101			

Note: Co-resident (No) = living but not co-residing grandmother; MGM = Maternal grandmother.

Similarly, there was a significant interaction between grandfather co-residence status and grandchild age (χ^2^_2_ = 23.30, *P* < 0.001), and again there was a significant difference between the interaction of grandchild age and no living grandfather and the interaction with no co-resident grandfather (β = −0.312 ± 0.066, *P* < 0.001; Table 2) – at younger ages, it appears (Figure 1b) that there was a beneficial effect of having a living grandfather who was not co-resident, but this difference was not apparent after infancy. Contrary to grandmother analysis where there were no significant interactions concerning co-residing grandmothers, there was a significant difference with the interaction of grandchild age and co-resident grandfather (β = −0.194 ± 0.071, *P* = 0.006). From Figure 1b, it appears that this difference was in opposite direction than previous one – detrimental with co-residence – but only in infancy. Thus, hypothesis 1 was partly supported. Also hypothesis 3 was partly supported since not co-residing grandmothers were beneficial for very young children whereas co-residing grandfathers were not.

**Table 2:** Generalized linear mixed-effect model outputs for grandchild survival by grandfather co-residence. Reference level for co-residence was “No living grandfather.” *N* is number of individuals

Fixed Effects	Estimates	Standard Error	*Z*	*P*	*N*
Intercept	2.368	0.209	11.31	<0.001	1796
Age	0.454	0.044	10.36	<0.001	
Co-resident (Yes)	0.049	0.120	0.41	0.684	1020
Co-resident (No)	0.315	0.116	2.72	0.007	1426
Living Siblings	1.170	0.039	29.95	<0.001	
Twin	−1.401	0.172	−8.15	<0.001	
Birth Order	−0.435	0.017	−25.13	<0.001	
Age * Co-resident (Yes)	−0.194	0.071	−2.74	0.006	
Age * Co-resident (No)	−0.312	0.066	−4.76	<0.001	
Random Effects	Variance	Standard Deviation			
MGF ID	<0.001	<0.001			
Birth Cohort	0.010	0.10			

Note: Co-resident (No) = living but not co-residing grandfather; MGF = Maternal grandfather.

**Figure 2:**
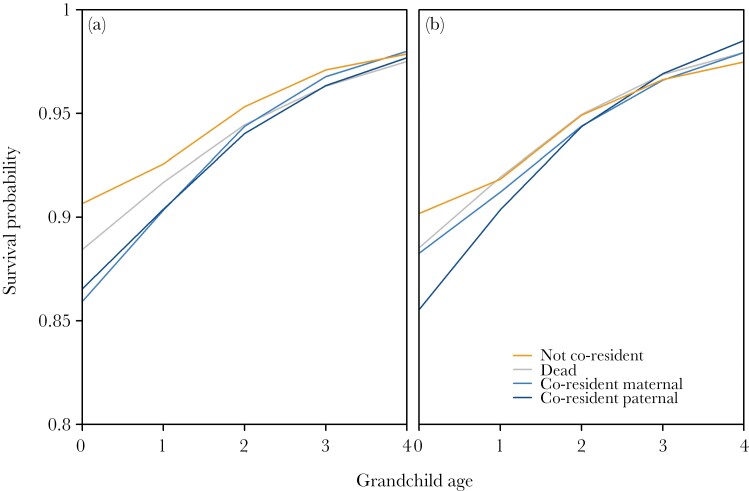
**Model-predicted grandchild survival by the interaction of grandparental co-residence and grandchild age for any grandparent, partitioned by grandmother lineage.** Orange = both grandmothers/grandfathers are not co-resident, grey = grandmothers/grandfathers are dead, lighter blue = maternal grandmother/grandfather is co-resident, darker blue = paternal grandmother/grandfather is co-resident. Lines are smoothed splines created with the *smooth.spline* function in R, with default parameter values. a) Grandmothers, b) grandfathers. For density distributions of predicted survival probabilities, see Figure S2.

**Figure 3:**
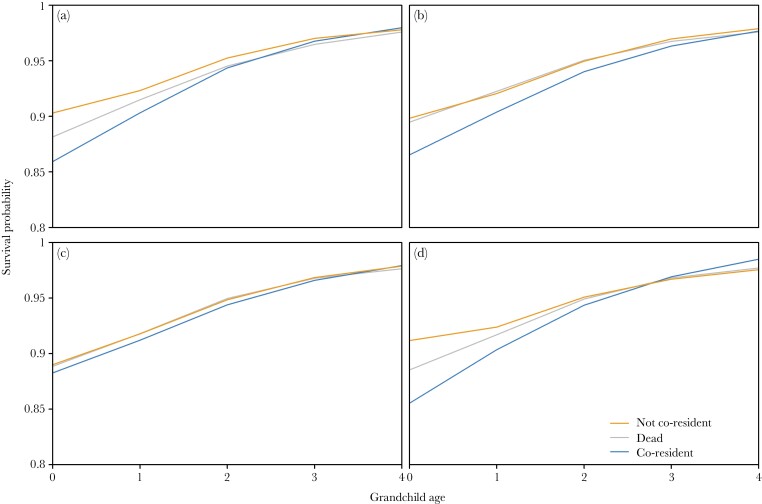
**Model-predicted grandchild survival by the interaction of grandparental co-residence and grandchild age for each grandparent.** Orange = grandparent not co-resident, grey = focal grandparent is dead, blue = focal grandparent is co-resident. Lines are smoothed splines created with the *smooth.spline* function in R, with default parameter values. a) Maternal grandmother, b) maternal grandfather, c) paternal grandmother, d) paternal grandfather. For density distributions of predicted survival probabilities, see Figure S3.

Hypotheses 2 predicted that, according to sex-specific reproductive strategies, the possible negative effect of having a co-residing grandparent should be more visible in the case of paternal grandparents. Though there was a significant interaction of partitioned co-residence and grandchild age (χ^2^_3_ = 8.23, *P* = 0.042; Figure 2a) and in the separate grandmaternal (χ^2^_2_ = 9.27, *P* = 0.010; Figure 3a) models, there was no detrimental effect of co-resident with either lineage as compared with no living grandmother/grandfather (Table 3). There were, however, higher survival probabilities of infants who were not living with either grandmother than those with no living grandmother (Figures 2a and 3a). The paternal grandmother-only model had no significant interaction with grandchild age (χ^2^_2_ = 4.12, *P* = 0.128; Figure 3c), indicating that co-residence with a paternal grandmother had no effect on survival.

**Table 3:** Generalized linear mixed-effect model outputs for grandchild survival by partitioned grandmother co-residence. Reference level for co-residence was “No living grandmother.” *N* is number of individuals

Model	Fixed Effects	Estimates	Standard Error	*Z*	*P*
Both	Intercept	2.290	0.219	10.48	<0.001
	Age	0.404	0.053	7.68	<0.001
	Co-resident (MGM)	0.021	0.191	0.11	0.915
	Co-resident (PGM)	0.093	0.148	0.63	0.532
	Co-resident (No)	0.250	0.121	2.06	0.039
	Living Siblings	1.151	0.038	30.05	<0.001
	Twin	−1.348	0.170	−7.93	<0.001
	Birth Order	−0.425	0.017	−25.11	<0.001
	Age * Co-resident (MGM)	−0.009	0.117	−0.07	0.941
	Age * Co-resident (PGM)	−0.103	0.085	−1.21	0.228
	Age * Co-resident (No)	−0.177	0.066	−2.69	0.007
MGM	Intercept	2.316	0.204	11.34	<0.001
	Age	0.374	0.040	9.38	<0.001
	Co-resident (MGM)	−0.009	0.177	−0.05	0.960
	Co-resident (No)	0.236	0.100	2.36	0.018
	Living Siblings	1.151	0.038	30.16	<0.001
	Twin	−1.353	0.170	−7.97	<0.001
	Birth Order	−0.423	0.017	−25.15	<0.001
	Age * Co-resident (MGM)	0.021	0.112	0.19	0.852
	Age * Co-resident (No)	−0.166	0.057	−2.91	0.004
PGM	Intercept	2.416	0.202	11.94	<0.001
	Age	0.349	0.039	8.92	<0.001
	Co-resident (PGM)	−0.027	0.130	−0.21	0.836
	Co-resident (No)	0.091	0.106	0.86	0.391
	Living Siblings	1.150	0.038	30.13	<0.001
	Twin	−1.344	0.170	−7.92	<0.001
	Birth Order	−0.427	0.017	−25.50	<0.001
	Age * Co-resident (PGM)	−0.046	0.078	−0.59	0.556
	Age * Co-resident (No)	−0.125	0.061	−2.04	0.042
	Random Effects	Variance	Standard Deviation		
Both	MGM ID	<0.001	<0.001		
	Birth Cohort	0.010	0.10		
MGM	MGM ID	<0.001	<0.001		
	Birth Cohort	0.011	0.105		
PGM	MGM ID	<0.001	<0.001		
	Birth Cohort	0.008	0.093		

Note: MGM = Maternal grandmother, PGM = Paternal grandmother, Co-resident (No) = living but not co-residing grandmother.

Partitioning grandfather co-residence by lineage showed similar results, with a significant interaction of grandchild age and partitioned grandfather co-residence (χ^2^_3_ = 25.08, *P* < 0.001; Figure 2b), and significant interactions in the separate lineage models (maternal χ^2^_2_ = 6.50, *P* = 0.039; paternal χ^2^_2_ = 16.58, *P* <0.001; Figure 3b and d). Again, there was no detrimental effect of co-residence with either lineage (i.e., survival probabilities were not lower than the baseline of no living grandfather; Table 4), only a somewhat beneficial effect in infancy to being not co-resident compared with having no living grandfather (Figures 2b and 3d). Note here that the effect is barely distinguishable in Figure 3b, suggesting that the effect for maternal grandfathers on grandchild survival likely has no real biological or sociological relevance.

**Table 4:** Generalized linear mixed-effect model outputs for grandchild survival by partitioned grandfather co-residence. Reference level for co-residence was “No living grandfather.” *N* is number of individuals

Model	Fixed Effects	Estimates	Standard Error	*Z*	*P*
Both	Intercept	2.356	0.210	11.22	<0.001
	Age	0.454	0.044	10.36	<0.001
	Co-resident (MGF)	0.145	0.224	0.65	0.516
	Co-resident (PGF)	0.021	0.156	0.13	0.894
	Co-resident (No)	0.247	0.108	2.29	0.022
	Living Siblings	1.172	0.039	29.94	<0.001
	Twin	−1.393	0.172	−8.09	<0.001
	Birth Order	−0.436	0.017	−25.16	<0.001
	Age * Co-resident (MGF)	−0.191	0.136	−1.41	0.159
	Age * Co-resident (PGF)	−0.110	0.100	−1.10	0.271
	Age * Co-resident (No)	−0.303	0.061	−4.97	<0.001
MGF	Intercept	2.401	0.204	11.76	<0.001
	Age	0.367	0.036	10.19	<0.001
	Co-resident (MGF)	0.079	0.219	0.36	0.718
	Co-resident (No)	0.112	0.102	1.10	0.271
	Living Siblings	1.157	0.039	29.90	<0.001
	Twin	−1.375	0.172	−8.02	<0.001
	Birth Order	−0.427	0.017	−25.09	<0.001
	Age * Co-resident (MGF)	−0.103	0.133	−0.77	0.439
	Age * Co-resident (No)	−0.152	0.060	−2.54	0.011
PGF	Intercept	2.365	0.198	11.93	<0.001
	Age	0.377	0.035	10.92	<0.001
	Co-resident (PGF)	−0.017	0.147	−0.11	0.909
	Co-resident (No)	0.325	0.116	2.79	0.005
	Living Siblings	1.160	0.039	29.90	<0.001
	Twin	−1.377	0.171	−8.04	<0.001
	Birth Order	−0.426	0.017	−25.19	<0.001
	Age * Co-resident (PGF)	−0.033	0.096	−0.35	0.730
	Age * Co-resident (No)	−0.276	0.067	−4.14	<0.001
	Random Effects	Variance	Standard Deviation		
Both	MGF ID	<0.001	<0.001		
	Birth Cohort	0.010	0.101		
MGF	MGF ID	<0.001	<0.001		
	Birth Cohort	0.011	0.106		
PGF	MGF ID	<0.001	<0.001		
	Birth Cohort	0.011	0.107		

Note: MGF = Maternal grandfather, PGF = Paternal grandfather, Co-resident (No) = living but not co-residing grandfather.

## DISCUSSION

This study investigated whether living in a three-generational family was associated with child survival in pre-industrial Finland. A major advance is that we were able to actually identify the co-residence status of a grandparent and grandchild and to see whether the potential effect was dependent on the age of the grandchild. Moreover, as we had data for co-residing paternal and maternal grandparents, it was possible to separate both the sex and lineage effects. Although almost three quarter of three-generational households in this population consisted of paternal grandparents, parents, and children, also a considerable proportion of three-generational households had maternal grandparents living with their child’s family. This is important because resource competition can exist between kin from either maternal or paternal side (Sear 2008).

We found that co-residing with a grandmother was not associated either positively or negatively with child survival in this sample as compared with not having a living grandmother at all and it did not make a difference whether the co-residing grandmother was paternal or maternal. In line with the grandmother hypothesis, having a living but not co-residing grandmother was, however, beneficial for very young grandchildren (age < 1) as compared with not having a living grandmother. Co-residing with a grandfather, in turn, was negatively associated with child survival during infancy as compared with not having a living grandfather although at the same time having not co-residing grandfather seemed to be beneficial as compared with not having a living grandfather. When looking at the survival associations and interaction with grandchild age and grandparent co-residence separately with maternal and paternal grandfather, the above-mentioned negative association turned into non-significant. The results, although not unequivocal, are in line with both the presence of resource competition (e.g., Strassmann and Garrard 2011) and grandmother effect (Hawkes et al. 1998) but as we did not find clear differences between lineages, the sex-specific reproductive strategies (Euler 2011) did not gain support.

Our results are partly in line with a previous work that has identified grandmaternal age and health as important factors for child survival (Chapman et al. 2019). In this particular study, we did not have the statistical power to address whether advanced age or declining grandparental health may mediate the association between grandparent presence and grandchild survival in co-resident households. These factors, however, may have played a role in the negative association of grandfather co-residence and grandchild age interaction and survival.

Negative associations related to grandfathers’ co-residence and positive associations related to grandmothers’ who were not co-residing with a grandchild were both dependent on grandchild age. We found significant co-residence status and grandchild age interaction which indicated that beneficial as well as detrimental effects were present only when grandchildren were very young (age < 1). Previous studies among the same population (Chapman et al. 2019; Lahdenperä et al. 2004) have also found positive associations, especially for maternal grandmother presence which applies, however, mainly to children between ages 2 to 5 whereas our results, restricted to a smaller sample with the information of co-residence status, applied to infants. In addition to resource competition between co-residing infant and grandfather, the detrimental effects for infant survival could be due to infectious deceases which are shown to be most severe for infants and older people (Glynn and Moss 2020).

We did not find any significant bias in our sample compared with the whole population, for instance, in whether the child’s family was landed or landless. However, due to data limitations we do not have exact information e.g., of the house sizes for our sample population which could be a more precise indicator for the wealth of the family instead of mere division to landed and landless. In our sample it was more common for landed than landless people to live in three generation households. Thus, three generational household were biased towards wealthier (however not by no means rich) population and it is not likely that the result were due to the selection into poorer households.

Although, of course, there are several major differences between three-generational households in pre-industrial agrarian population and in industrialized or contemporary societies (and one cannot make comparisons between them lightly), it is interesting to see that co-residing grandparents can have harmful influence for children in both settings. One such difference is that three-generational households in pre-industrial Finland were common among reasonably wealthy farmers, those who usually owned their farm and thus had more resources than the poorest agrarian population, whereas nuclear families were more common among poorer groups such as landless, crafters or tenant farmers. (Moring 2003). In contemporary societies (Pilkauskas and Martinson 2014) and in historic industrialized societies (Hacker et al. 2021; Willführ et al. 2022), on the contrary, three-generational households are rare among well off people and more common among people on lower socioeconomic standings. Thus, the reason for negative grandparent effects on fertility and child survival found in previous studies could be caused by selection and not by a causal grandparent effect per se (Hacker et al. 2021; Willführ et al. 2022). The situation is different in our study population where three-generation families were more common for well off people and thus the selection is opposite, meaning that three-generation families consist of people on higher socioeconomic standings. Especially due to these disparities an interesting question that should be studied more is whether co-residing grandparents actually have a causal effect on grandchild wellbeing or whether the association is due to selectivity.

Also the mechanisms behind negative associations between grandparental co-residence and grandchild survival require more investigation. What comes to resource competition, the detrimental effects could be either as a consequence of competition for care of a middle generation or for other resources in the household. Unfortunately, we were not able to distinguish between these two with the current data. Moreover, with larger data resource competition hypothesis could be studied more precisely in relation to hunger years or other crop failures.

Here, we have studied the association between grandparental co-residence and child survival in childhood. Our results have important implications for studying in the future whether childhood co-residence with grandparents might have any long-term life-history consequences for children. Whilst we focused here on possible resource competition affecting grandchild survival, competition can act in both directions of the intergenerational relationship. Thus, future studies should also address whether there are costs or benefits of co-residence for the older generation, in order to build a clearer picture of the evolutionary costs and benefits of family living.

## Supplementary Material

arad013_suppl_Supplementary_Material

## DATA ACCESSIBILITY

Analyses reported in this article can be reproduced using the data provided by Chapman et al. (2023).
