# Supplementary material for: Grandparental co-residence and grandchild survival: the role of resource competition in a pre-industrial population
Source: Behav Ecol. 2023 Mar 27;34(3):446–56. doi: 10.1093/beheco/arad013 (PMC10183204; doi:10.1093/beheco/arad013)

**Supplementary material**

**Figure captions for supplementary figures 1, 2, and 3.**

**Figure S1. Model-predicted grandchild survival by the interaction of grandparental co-residence and grandchild age for grandmothers/grandfathers.** Grey = grandmothers/grandfathers are dead, blue = a grandmother/grandfather is co-resident, orange = both grandmothers/grandfathers are not co-resident. Lines are smoothed splines created with the *smooth.spline* function in R, with default parameter values. Violin plots show the density distributions of predicted survival probabilities (bandwidth = 0.02). A) Grandmothers, B) grandfathers.


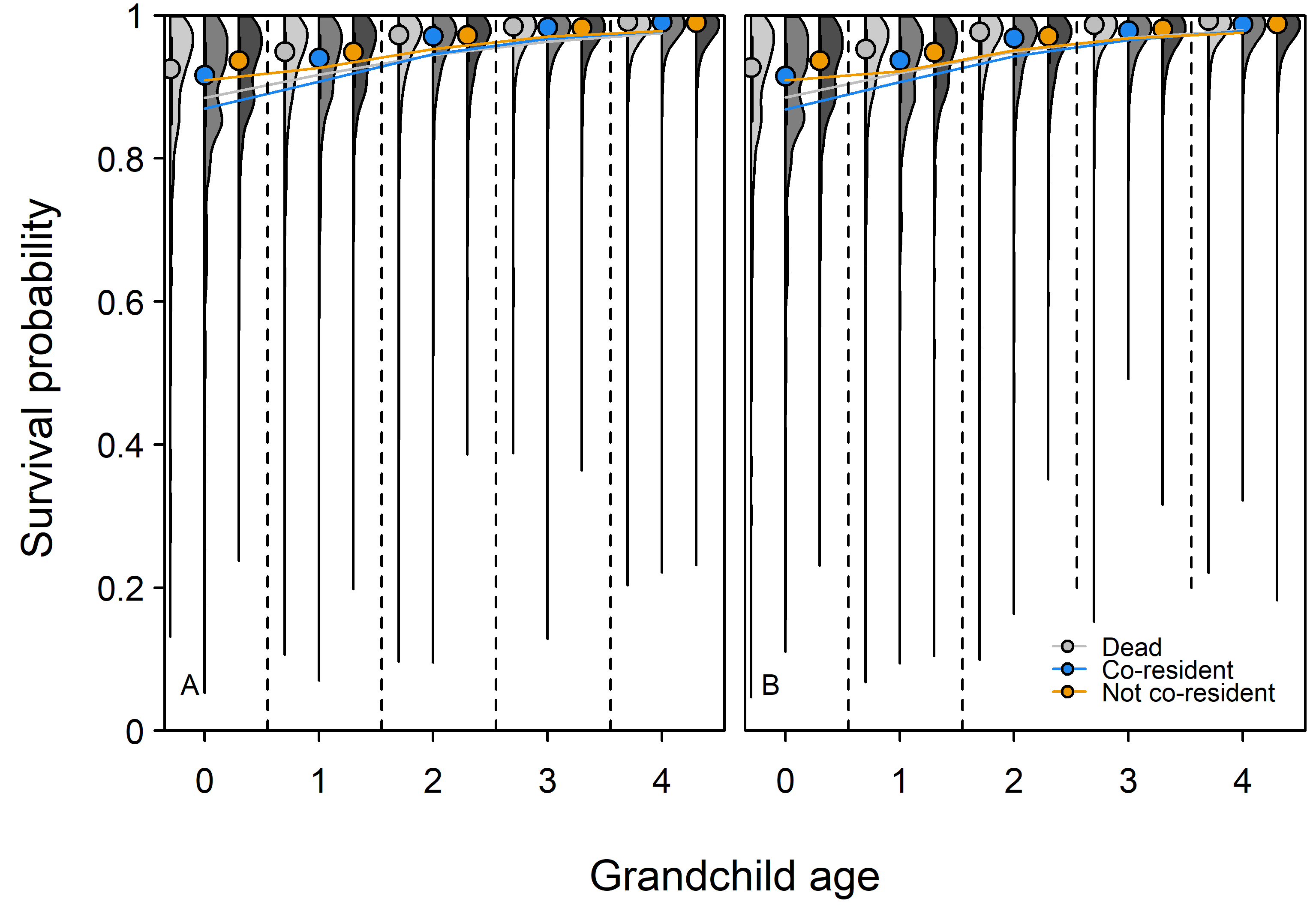


**Figure S2. Model-predicted grandchild survival by the interaction of grandparental co-residence and grandchild age for any grandparent, partitioned by grandmother lineage.** Grey = grandmothers/grandfathers are dead, lighter blue = maternal grandmother/grandfather is co-resident, darker blue = paternal grandmother/grandfather is co-resident, orange = both grandmothers/grandfathers are not co-resident. Lines are smoothed splines created with the *smooth.spline* function in R, with default parameter values. Violin plots show the density distributions of predicted survival probabilities (bandwidth = 0.02). A) Grandmothers, B) grandfathers.


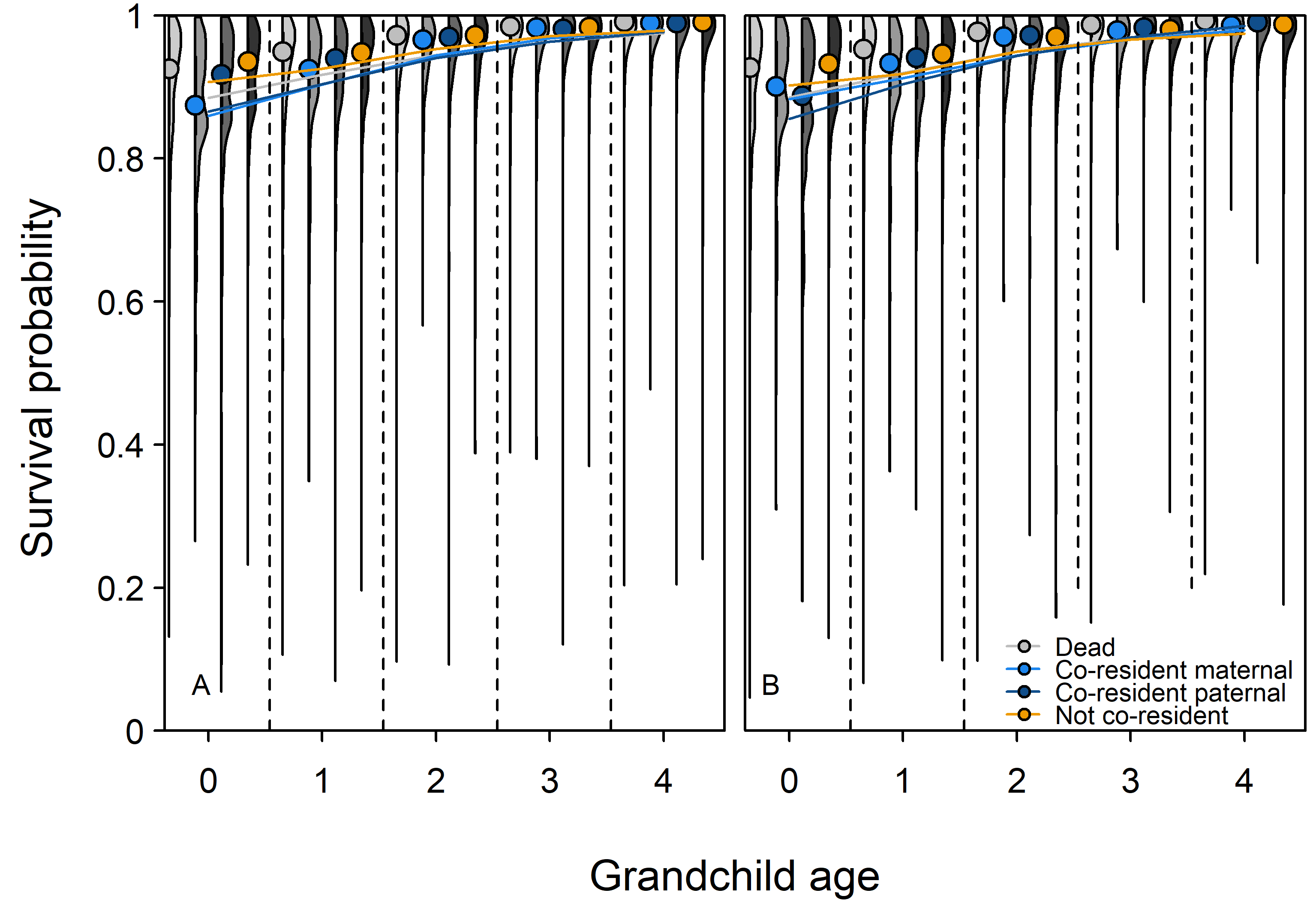


**Figure S3. Model-predicted grandchild survival by the interaction of grandparental co-residence and grandchild age for each grandparent.** Grey = focal grandparent is dead, blue = focal grandparent is co-resident, orange = grandparent not co-resident. Lines are smoothed splines created with the *smooth.spline* function in R, with default parameter values. Violin plots show the density distributions of predicted survival probabilities (bandwidth = 0.02). A) Maternal grandmother, B) maternal grandfather, C) paternal grandmother, D) paternal grandfather.


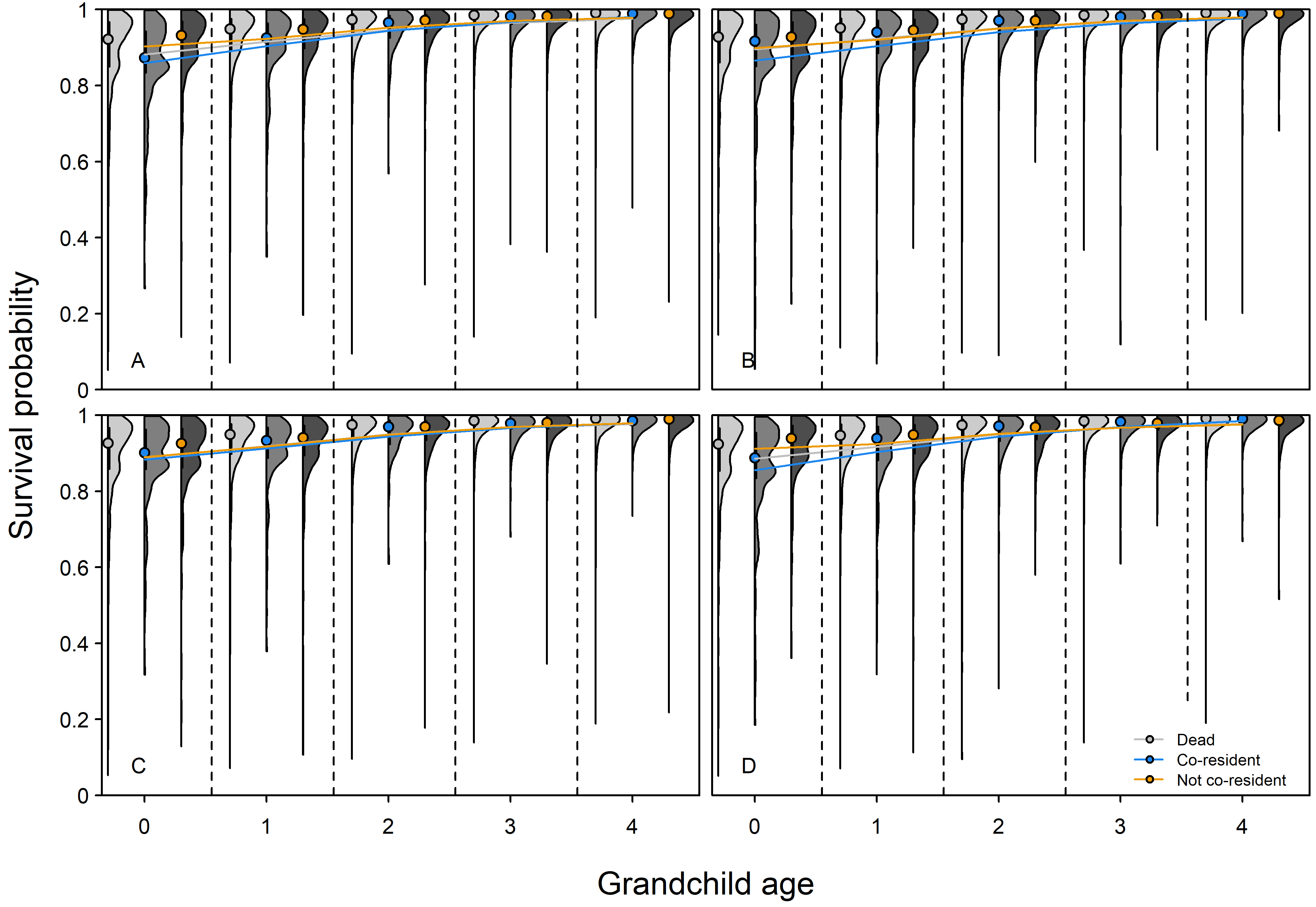

Supplement: arad013_suppl_Supplementary_Material [file arad013_suppl_supplementary_material.docx]
